# Whooping Cough

**Published:** 1861-03

**Authors:** 


### Whooping Cough.

For this disease, Dr. Benson, of the Louisville *Journal*, recommends the following:

| | |
|---|---|
| ℞.—Acid Hynrocyan, | gtt vi. |
| Ext. Belladonna, | grs. ii. |
| Tinct. Opii Camph., | ʒ iii. |
| Syr. Bals. Tolu, | ℥ ii· |
| Aquæ Font, | ℥ iii. |

Misce. S. One teaspoonful four times a day.

